# Elevation of serum neurofilament light-chain levels disclose possible occult progressive multifocal leukoencephalopathy and immune reconstitution syndrome in a patient receiving ozanimod: a case report

**DOI:** 10.3389/fimmu.2024.1465678

**Published:** 2024-10-22

**Authors:** Carlos Quintanilla-Bordás, David Gorriz, Laura Cubas-Núñez, Jéssica Castillo-Villalba, Joan Carreres-Polo, Bonaventura Casanova, Francisco Carlos Pérez-Miralles

**Affiliations:** ^1^ Neuroimmunology Unit, Polytechnic and University Hospital La Fe, València, Spain; ^2^ Radiology Department, Polytechnic and University Hospital La Fe, València, Spain

**Keywords:** neurofilament light (NfL) chain, ozanimod (PubChem CID: 52938427), progressive multifocal leukoencephalopathy (PML), immune reconstitution inflammatory syndrome (IRIS), multiple sclerosis

## Abstract

**Background:**

We report the first case of findings suggestive of progressive multifocal leukoencephalopathy and immune restitution syndrome (PML-IRIS) in a patient with multiple sclerosis receiving ozanimod preceded by an unexpected increase in the serum neurofilament light-chain (sNfL) levels.

**Case report:**

A 57-year-old-woman treated with ozanimod for the last 8 years presented, during surveillance MRI, with findings compatible with PML-IRIS. Overt clinical symptoms were absent. sNfL levels increased 4 months earlier and peaked at presentation. Lymphocyte count reached nadir of 330/mL at 8 months earlier.

**Conclusion:**

The case illustrates the utility of sNfL levels for PML surveillance in patients receiving immunosuppressors.

## Background

Progressive multifocal leukoencephalopathy (PML) is a severe destructive opportunistic infection of the central nervous system characterized by rapid progression of neurological disability due to axonal destruction. Serum neurofilament light chain (sNfL), a biomarker of neuroaxonal injury, has shown to elevate to very high levels in this setting.

Ozanimod is an oral spingosine-1-receptor (S1PR) modulator used to treat relapsing forms of multiple sclerosis (MS) and ulcerative colitis. As other disease-modifying treatments (DMT) for MS, S1PR modulators have been associated with infections, including PML. The drug binds to S1PR on the surface of lymphocytes, preventing egression from lymph nodes and, thus, reducing migration of inflammatory cells to the central nervous system. Since first clinical trials and approval in 2020, the manufacturer estimates an exposition of 40,000 patient-years wordwide as of April 2023. So far, only one case of PML has been reported (data available by Bristol-Myers Squibb) (2).

The case presented herein is, to our knowledge, the first case of a possible PML simultaneous with immune restitution syndrome (PML-IRIS) in a patient ongoing treatment with ozanimod, which was disclosed by an unexpected elevation of sNfL levels.

## Case report

**Figure 1:**
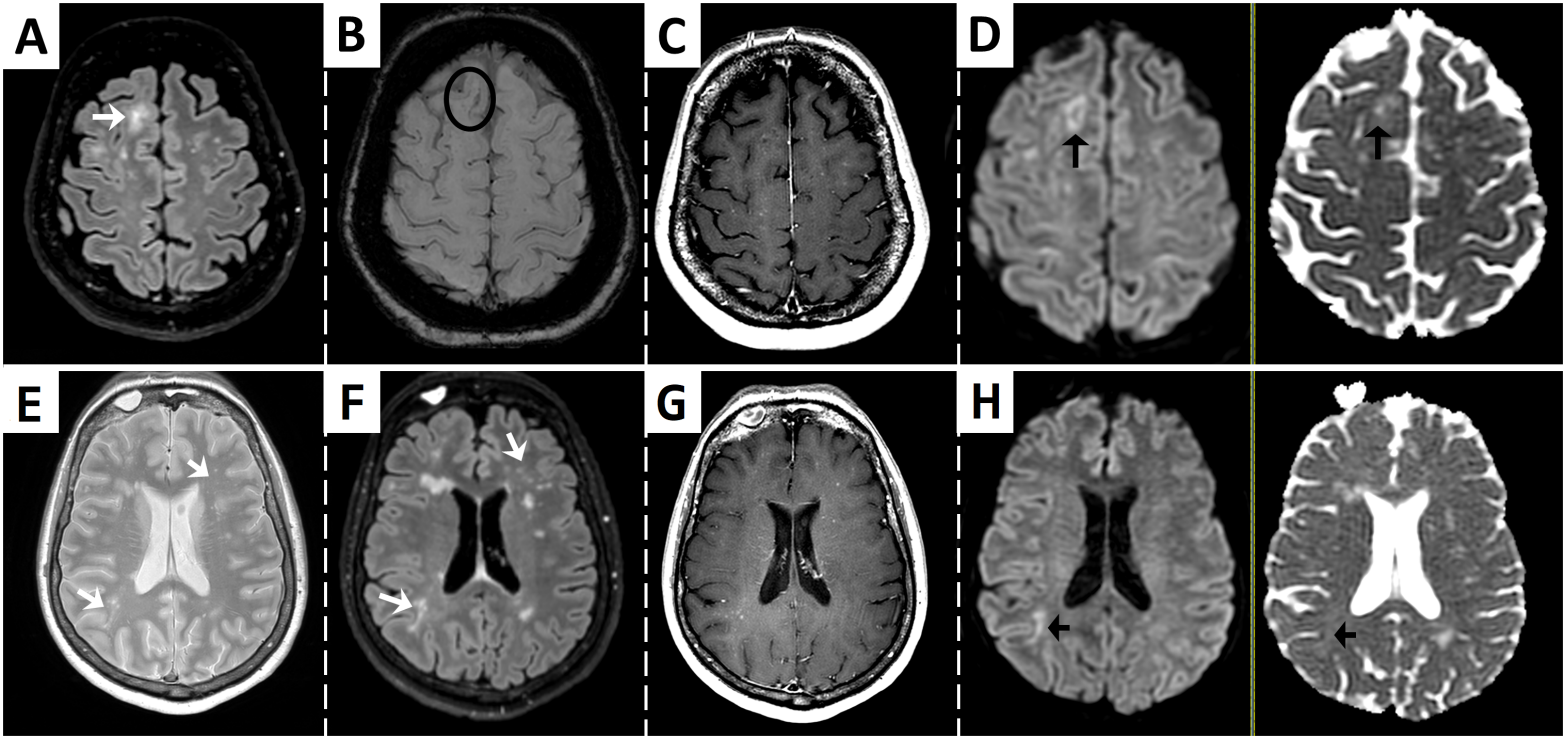
Brain MRI at presentation showing findings suggestive of multifocal progressive leukoencephalopathy and immune reconstitution inflammatory syndrome. Fluid-attenuated inversion recovery (FLAIR) sequence shows an ill-defined subcortical and juxtacortical hyperintense lesion [arrow in **(A)**], with a paramagnetic rim adjacent to the cortex in susceptibility-weighed imaging suggestive of iron deposition [circle in **(B)**]. T1-weighted post-gadolinium sequence reveals punctate hyperintensities that suggest enhancement or perivascular spaces **(C)**. Diffusion-weighed (DWI) imaging shows a hypointense core surrounded by restriction at the periphery [left arrow in **(D)**]. ADC map demonstrates variable low ADC values of similar intensity to surrounding normal white matter [right arrow in **(D)**]. Other ill-defined and punctate lesions can be seen in T2 and FLAIR images (arrows in **(E, F)**, respectively), with punctate enhancement [T1-weighted post-gadolinum image in **(G)**] and restricted diffusion of a right subcortical lesion [arrows in **(H)**].

A 57-year-old woman with relapsing remitting MS treated with ozanimod for the last 8 years presented, during routine follow-up MRI for surveillance due to marked sNfL level elevation, with findings suggestive of PML and IRIS (**Figure 1**). Her medical history was relevant for hypertension and depression. Five months prior, she suffered from mild SARS-CoV-2 infection with systemic and upper respiratory tract symptoms. She received ozanimod as her first DMT after diagnosis 10 years before, initially as part of a clinical trial, and then during routine clinical practice. Ever since, her disease had remained stable with no changes in expanded disability status scale (EDSS) and no signs of activity in yearly brain MRIs. Her baseline EDSS was 2.5, and her neurological exam was remarkable for sensory loss in her left arm, hyperreflexia, and mild decrease of mentation. In retrospect, the patient referred worsening of mentation months prior that could not be confirmed on neurological examination. At the time of MRI scan showing findings suggestive of PML, she had received ozanimod uninterruptedly for 115 months. The patient was admitted for rapid workup. Commercial real-time polymerase chain reaction (PCR; JCV ELITe MGB^®^ Kit, ELITechGroup S.p.A, Torino, Italy) of cerebrospinal fluid (CSF) was positive at 34 copies/mL, which was under the limit of the detection of the technique, and, therefore, was rendered negative by the laboratory. Repeated lumbar puncture 2 weeks later was also negative for JCV. CSF cytobiochemistry was unremarkable (glucose, 53 mg/dL; proteins, 34.8 mg/dL; and 3 leukocytes/μL). HIV serology was negative. Her absolute lymphocyte count (ALC) was 515/μL, 500/μL, and 330/μL (nadir) at presentation, 4 and 8 months, respectively, prior to MRI findings. She had performed ALC every 4 months, and all ALC ranged between 330 and 850 during the last 3 years. The remaining cell blood count and extensive biochemistry, including Immunoglobulin G (IgG), Immunoglobulin M (IgM), and Immunoglobulin A (IgA) levels, were within normal range.

**Figure 2:**
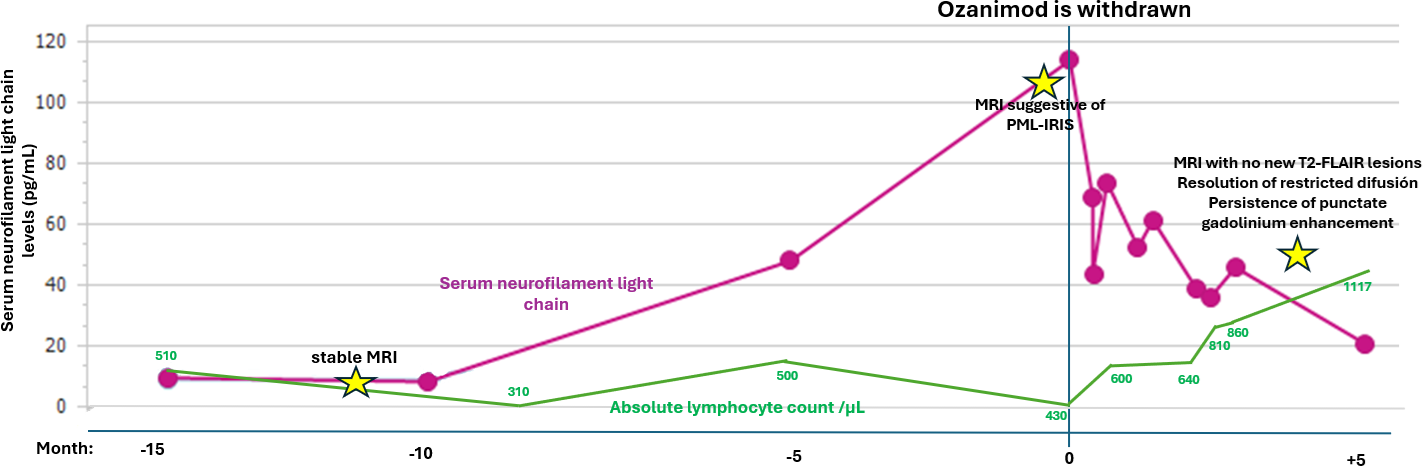
Timeline of events in relation to absolute lymphocyte count and serum neurofilament light-chain levels. PML, progressive multifocal leukoencephalopathy; IRIS, immune reconstitution inflammatory syndrome.

sNfL levels were determined every 4 months as part of clinical routine. Baseline sNfL levels in the year prior ranged between 8.5 pg/mL and 9.5 pg/mL but, 4 months before her levels rose to 47.9 pg/mL, peaked at 113.7 pg/mL at presentation and then descended to 43.4 pg/mL 2 weeks later. Retrospective revision of yearly brain MRIs confirmed no previous lesions suggestive of PML. A timeline of events of sNfL and ALC is shown in **Figure 2**.

Upon admission, the patient discontinued ozanimod and started treatment with oral mirtazapine 15 mg daily and a single dose of intravenous cidofovir 5 mg/kg body weight. Follow-up MRI scan performed 2 weeks later did not show significant changes. Subsequent MRI scan performed 24 days later showed, for the first time, absence of new gadolinium, enhancing lesions and similar T2/Flair lesions. Four months later, MRI shows no changes in T2/FLAIR lesions, resolution of restricted diffusion foci, and persistence of punctate gadolinium enhancement. She remains clinically stable, and the neurological exam has not shown any significant changes. Alternative therapy with fortnightly IM pegylated interferon β was initiated.

## Discussion

We describe a 57-year-old woman treated with ozanimod for 10 years, without other known risk factors such as prior immunosuppression or exposure to other DMTs, who presented with findings suggestive of PML-IRIS detected brain MRI performed after an unexpected rise in sNfL levels. Despite highly suggestive MRI findings and compatible clinical presentation, PCR positivity was below the limit of detection of the technique, and, therefore, the presence of JC virus DNA in CSF could not be confirmed. Hence, we cannot rule out completely breakthrough MS radiographic activity, although we point out that this is very unlikely given the radiographic appearance and extreme elevation of sNfL levels.

Asymptomatic or paucisymptomatic PML are increasingly recognized with growing number of patients treated with DMTs. Several MRI findings such as the presence punctate lesions in the vicinity of a new lesion (a.k.a. milky way sign, as seen in **Figure 1**) have proven extremely specific of PML (3). The presence of IRIS in this setting further increases specificity to the diagnosis. In this circumstance, MRI is the cornerstone of diagnosis, as several MRI findings have been proved extremely specific of PML (see **Figure 1**), such as the presence punctate lesions in the vicinity of a new lesion (a.k.a. milky way sign) (3). Our case also presented small punctate Gd enhancement within and outside of the main lesions, which is thought to correlate with histological findings of T- and B-cell infiltration due to partial immune reconstitution within perivascular spaces (4, 5). The fact that the patient presented with PML while ongoing treatment with ozanimod may explain the presence of partial IRIS. The patient also experienced an elevation of sNfL (a biomarker of axonal injury) from 9.5 pg/mL at baseline to 5-fold 4 months prior and to 12-fold at the time of diagnosis. This is in accordance with previous studies that show that sNfL increases more than 10-fold during PML and until IRIS onset. Such extremely high levels of sNfL are rarely seen are in MS and have shown to discriminate between PML and relapses. Such extremely high levels of sNfL are rarely seen are in MS and have shown to discriminate between PML and relapses (1, 6). However, PCR of CSF may yield false-negative results, especially in mild cases or when IRIS is present. Thus, a negative PCR in this setting should not discard PML. In such cases, biopsy becomes critical to establish diagnosis. Because our patient had minimal symptoms, the risk-benefit ratio of performing a biopsy was considered unfavorable. However, according to proposed criteria, diagnosis of PML cannot be established in an asymptomatic patient without a positive PCR in CSF or biopsy, leading to no diagnosis despite that it may evolve to a potentially lethal condition (7). In light of current biomarkers, we propose that criteria should be revised to include the presence of highly specific MRI findings accompanied by extreme sNfL elevation in the category of “possible PML”.

This report represents the first case of possible PML-IRIS and the second case of PML while on treatment with ozanimod, after a 46-year-old woman with symptomatic PML following treatment for 4 years, treated previously with interferon β-1a and an ALC of 500/μL (2). Our case differs in the paucity of symptoms and that it presented simultaneously with signs of IRIS (PML-IRIS) despite ongoing treatment. Although IRIS occurs usually weeks or months after cessation of the immunosuppressive treatment, another case of PML-IRIS while ongoing treatment with a S1PR modulator has also been reported (8). Months prior to presentation, our patient also suffered of mild SARS-CoV-2 infection, which has been related to PML infection too (9, 10), although the causality of these events remains speculative. The case reminds the necessity of surveillance for PML in patients with newer S1PR modulators, bearing in mind that overt clinical findings may not be present. The utility of sNfL should be underscored as our patient exhibited a striking increase in sNfL months prior to MRI findings allowing for a prompt diagnosis.

## Data Availability

The datasets presented in this article are not readily available because of ethical and privacy restrictions. Requests to access the datasets should be directed to the corresponding author/s.
